# Comprehensive Assessment of Local and Exotic Sorghum Genotypes for Forage Production and Quality Under Drought Conditions

**DOI:** 10.1155/sci5/9158280

**Published:** 2025-11-28

**Authors:** Zeba Ali, Amir Bibi, Rana Muhammad Atif, Muhammad Ashfaq Wahid

**Affiliations:** ^1^Department of Plant Breeding and Genetics, University of Agriculture, Faisalabad, Pakistan; ^2^Department of Agronomy, University of Agriculture, Faisalabad, Pakistan

**Keywords:** genotype, hydrogen cyanide, hydroponics, principal component analysis, sorghum fodder, yield

## Abstract

Sorghum (*Sorghum bicolor* L.), locally known as jowar, is a vital summer fodder crop in Pakistan, significantly contributing to livestock sustenance. However, drought stress poses a critical challenge by reducing plant biomass and elevating hydrogen cyanide (HCN) content, a toxic antiquality component that endangers livestock health. This study aimed to identify sorghum genotypes with improved fodder yield and reduced HCN content under drought stress. Seventy diverse genotypes were evaluated in a hydroponic system under three polyethylene glycol (PEG) levels (0%, 5%, and 10%) in a two-factor factorial experiment arranged in a completely randomized design (CRD). Analysis of variance (ANOVA) revealed highly significant (*p* < 0.05) genotype, treatment, and genotype × treatment interaction effects across all measured traits, indicating considerable genetic variability in drought responses. Drought stress significantly increased root length (RL) (3.2–13.2 cm) and decreased several morphological traits including shoot length (SL), shoot fresh and dry weights (SFW and SDW), and chlorophyll (23.4–42.8 μg cm^−2^) and fodder quality traits including crude protein (CP) (15.4%–24.1%) and crude fiber (CF). Principal component analysis (PCA) explained 72.4% of the total variance in the first three components, identifying SDW, SFW, RL, and SL as key contributors to drought tolerance. Correlation analysis revealed significant positive and negative correlations among the traits under all normal and drought conditions. Despite these reductions, genotypes such as Sorg-60, Sorg-66, and Sorg-7 showed superior performance in both biomass and quality traits, while Sorg-53 and Sorg-56 exhibited high sensitivity to drought. Based on PCA biplot positioning and trait performance, 20 genotypes (10 highly tolerant and 10 highly sensitive) were selected for field evaluation under normal and drought conditions using a randomized complete block design (RCBD). Morphological, physiological, and fodder quality traits showed comparatively low reduction under drought conditions in tolerant genotype compared to drought-sensitive genotypes. Statistical analyses supported the findings and highlighted promising genotypes for use in future sorghum breeding programs aimed at enhancing forage yield and nutritional safety under water-limited environments.

## 1. Introduction

The livestock industry holds a significant place within the socioeconomic framework of Pakistan [1]. Its pivotal role in employment generation, food security, and income diversification, particularly in rural contexts, cannot be overstated [2]. With an annual milk production of around 50 million tons, the dairy subsector meets domestic demand and supports livelihoods by generating employment and income for smallholder farmers [3].

Ensuring the well-being and productivity of livestock rely upon a multidimensional approach, with one key aspect being the provision of optimal nutrition through forage sources. The challenge, however, lies in cultivating fodders that can withstand the harsh realities of environmental stressors, particularly drought conditions, while providing abundant yields and superior quality [4]. Sorghum, a resilient and versatile crop, gained significant attention due to its potential to thrive in arid and semiarid regions, making it an invaluable candidate for fodder production under challenging climatic circumstances [5].

Pakistan's climatic and edaphic conditions are highly conducive to sorghum cultivation. However, poor crop management practices such as the use of low-yielding varieties and reduced plant population densities significantly limit forage biomass production [6]. Apart from these factors, high hydrogen cyanide (HCN) content under drought stress and insufficient fertilizer application are other major factors that limit the fodder production of sorghum [7].

HCN contents in sorghum vary greatly with the growth stages of the plant as well as environmental factors, including abiotic stresses, that is, frost, drought, and genotype. Any environmental stress (biotic or abiotic stress) can cause an increase in HCN toxicity as these affect the normal growth patterns of sorghum plants [8]. It is considered one of the most harmful factors that cause a significant reduction in crop yield and an increase in HCN content [9].

Drought is one of the main abiotic stresses that severely impacts sorghum growth and causes a reduction in fodder yield and nutritional quality [10]. This issue is particularly critical in regions like Pakistan, where factors such as delayed sowing and inadequate nutrient application further exacerbate the problem [11]. It severely disrupts cellular homeostasis and impacts fodder palatability and nutritional quality by causing a decrease in relative water content (RWC) in leaves [12]. HCN levels can rise significantly under drought stress and cause asphyxiation in livestock [13]. Consumption of sorghum with HCN concentrations beyond the safe limit of 200 ppm can cause severe toxicity in animals, showing serious symptoms such as respiratory distress, convulsions, and even death [14, 15].

Addressing these challenges requires a well-planned approach to the development of drought-tolerant sorghum genotypes with low HCN content and better nutritional quality. Breeding programs mainly focus on selecting parent genotypes with high yield, drought resilience, and minimal HCN toxicity [16]. The development of drought-tolerant sorghum varieties is crucial in the current climatic adversity. While several studies have examined either the drought tolerance or the HCN content of sorghum, few have integrated these aspects across a wide panel of genotypes. The present work is unique in that it evaluates 70 sorghum genotypes simultaneously for morphological and biochemical traits under both normal and drought-stress conditions. This comprehensive approach not only identifies drought-resilient and high-yielding genotypes but also ensures the selection of those with safer HCN levels and improved nutritional quality, thereby directly supporting breeding programs with dual goals of productivity and livestock safety. The objective of this study was to identify and evaluate sorghum genotypes for future breeding programs based on their morphological and biochemical traits under drought stress. This study will contribute to the development of improved sorghum varieties that can enhance livestock productivity while mitigating the risks associated with HCN toxicity.

## 2. Materials and Methods

### 2.1. Collection of Germplasm

Seventy genotypes (Supporting Table 1), including elite lines and available cultivars, were collected from USDA-ARS, USA; Fodder Research Institute (FRI), Sargodha; Fodder Research Institute, Ayub Agriculture Research Institute (AARI), Faisalabad; and Department of Plant Breeding and Genetics, University of Agriculture (UAF), Faisalabad, Pakistan.

### 2.2. Screening of Germplasm at the Seedling Stage Under Drought Stress

The experiment was performed in the wire house of the Department of Plant Breeding and Genetics, University of Agriculture, Faisalabad. Seventy genotypes, including elite lines and available cultivars of sorghum, were sown in polythene bags filled with soil. One-week-old seedlings were shifted to hydroponic conditions. One seedling per thermophore sheet hole was transplanted in a tin container (1.52 × 0.91 m) using an aerated half-strength Hoagland nutritional solution [17]. Tap water was used as a control (EC = 0.22 dSm^−1^). To maintain the ideal level of aeration in each tin container, air pumps were used. A two-factor factorial experiment was conducted in a CRD with 3 replications, and 3 treatments of PEG 6000 (0%, 5%, and 10%) were used, and data were recorded after 3 days of transplanting in aqueous media. A CRD is an experimental design in which all treatments are assigned to experimental units entirely at random, giving each unit an equal chance of receiving any treatment. This design is particularly suitable when experimental units are relatively homogeneous, as it minimizes bias and ensures that observed differences among treatments are primarily due to treatment effects. In the present study, CRD was selected because the conditions were kept uniform, and it allowed us to efficiently evaluate multiple genotypes under controlled drought treatments with adequate replication. The electrical conductivity (EC) of each drought stress level was maintained till the completion of the experiment. Each genotype was analyzed for some morphological traits, that is, RL (cm), SL (cm), fresh shoot weight (g), fresh root weight (g), dry root weight (g), dry shoot weight (g), and physiological traits, that is, chlorophyll content (CC) (μg cm^−2^), RWC (%), and total digestible nutrients (TDN). The studied morphological traits were selected because they represent early growth responses to drought stress. In addition, some biochemical traits, that is, acid detergent fibers (ADFs) (%), neutral detergent fibers (NDF) (%), crude protein (%), crude fibers (%), ash contents (%), and proline content (PC) (μmol mg^−1^ FW), were also calculated from frozen leaf tissues. HCN was also determined using a picric acid paper test at 4 weeks of the seedling stage. For this purpose, fresh tissues of each genotype from control and drought levels were collected in polythene bags and stored at −80°C refrigerator available in Transformation Lab, Center of Advanced Studies, UAF.

### 2.3. Determination of Total Cyanide in Leaves

A 100 g sorghum sample was finely chopped using scissors. A buffer paper was placed at the bottom of a plastic bottle, and the chopped sample was added on top. Approximately 1 mL of distilled water was introduced into the bottle. A yellow indicator paper, adhered to a plastic strip, was inserted into the bottle, and the cap was securely tightened to prevent the escape of cyanide gas. The samples were left to stand at room temperature for 24 h. The following day, the indicator papers were transferred to test tubes, and 5 mL of distilled water was added to each tube. The absorbance of the resulting solution was measured at 510 nm using a spectrophotometer. The total cyanide content was calculated in parts per million (ppm) using the formula as follows [18]:

(1) $$\begin{array}{c} t\text{otal cyanide content }\left(\text{ppm}\right)=\text{absorbance}\times 396. \end{array}$$

### 2.4. Evaluation of Selected Genotypes in the Field Conditions Under Drought Conditions

Twenty genotypes were selected based on the abovementioned parameters. Ten genotypes have high fodder yield, good quality, drought tolerance, and low HCN, 10 have lower fodder yield, low quality, drought susceptibility, and high HCN. Selected genotypes were evaluated in fields at the research area of the Department of Plant Breeding and Genetics, University of Agriculture, Faisalabad. A two-factor factorial experiment was laid out in a RCBD with three replications and two factors (normal, drought) in the field. Throughout the growing period, the plants were exposed to typical semiarid summer conditions. Average daily temperatures ranged from 35°C to 37°C, with daytime highs reaching up to 43°C. Relative humidity increased from approximately 18% in late June to around 51% in July due to the onset of the monsoon. The experimental site received a total rainfall of approximately 83.76 mm during the growing season, with most precipitation occurring in July. Light availability was high, with an estimated 297 h of sunshine recorded in July, ensuring adequate photosynthetic activity. The soil at the site was classified as sandy loam. Five plants from each genotype were selected at random before panicle emergence. Data were recorded before panicle emergence for forage yield, drought, HCN, and quality parameters which included plant height (PH) (cm), number of leaves (NOL)/plant, leaf area (LA) (cm^2^), green forage yield (GFY) (g), ADF (%), NDF (%), CP (%), TDN (%), leaf water contents (%), CCs (μg cm^−2^), PCs (μmol mg^−1^ FW), crude fiber content, ash contents (%) and HCN.

### 2.5. Statistical Analysis

All statistical analyses were performed using R version 4.2.2 in RStudio. ANOVA was conducted using the rstatix package to determine the significance of differences among treatments and genotypes. PCA analysis was carried out using the FactoMineR and factoextra, ggplot2 packages, which provided visualization and dimensional reduction of multivariate trait data to identify the most influential traits contributing to drought tolerance. In addition, Pearson correlation analysis was performed to evaluate the strength and direction of linear relationships among morphological and biochemical traits, helping to identify associations useful for selection in breeding programs.

## 3. Results

### 3.1. ANOVA

ANOVA depicted significant differences among 70 genotypes of sorghum at the seedling stage for fodder quality, yield, and physiological parameters related to drought under observation (Table 1). Variances of all parameters under study exhibited divergent responses against drought stress. Genotypes and genotype × treatment interaction revealed significant differences at (*p* ≤ 0.05) for all the traits under study. The highest variability among the traits was observed in treatments (T), with exceptionally high mean squares observed for SL, ADF, NDF and HCN. It indicated that the applied treatments exerted a profound effect on plant growth, biomass, and biochemical compositions. Genotypic effects (G) were also highly significant for all the traits, indicating the presence of high genetic variability among the studied genotypes, whereas G × T was significant but comparatively contributed less to the total variation. It suggests that the magnitude of interaction effects was smaller relative to the main effect.

### 3.2. Effect of Treatments on Morphological Traits

Significant variations were observed in morphological traits across all the genotypes and treatments. RL increased with the increase in drought stress level. For example, the RL remained 15.66 cm (mean) under T1, 18.83 cm in T2, and 22.81 cm in T3, corresponding to overall 45.6% increase from T1 to T3. Under controlled conditions (T1), the lowest RL was observed in Sorg-60 (3.17 cm) and Sorg-29 (3.9 cm), and the heights were recorded in Sorg-66 (29.1 cm), Sorg-69 (25.4 cm), and Sorg-70 (24.9 cm) (Supporting Table 2). The maximum increase in RL under T2 was recorded in Sorg-66, Sorg-69, and Sorg-70 by 31.3, 29.7, and 28.4 cm, respectively. Similarly, under T3, the maximum increase was recorded in Sorg-66, Sorg-70, Sorg-64, and Sorg-10 by 35.8, 35.5, 31, and 31.1 cm, respectively (Supporting Table 2).

In contrast, SL decreased significantly across all genotypes with increase in drought stress level. The maximum increase in SL under controlled conditions was recorded in Sorg-66, Sorg-43, and Sorg-39 by 46.5 cm, 42.4 m, and 40 cm, respectively, while the lowest in Sorg-52 (13.6 cm), Sorg-48 (18 cm), and Sorg-33 (17.4 cm). Under T2, the highest decrease in SL was recorded in Sorg-6, Sorg-26, and Sorg-56 by 12.5, 16, and 16.1 cm, respectively (Supporting Table 2). Under T3 shoot, length declined more sharply as compared to T2 with the highest decrease in Sorg-12 by 8.6 cm, Sorg-68 by 11.6 cm, and Sorg-1 by 11.3 cm.

Root fresh weight (RFW) consistently increased with the increase in treatment level. Sorg-41, Sorg-16, and Sorg-14 showed maximum gain under T1 by 0.177, 0.174, and 0.165 g, respectively, whereas under T2, the maximum gain in RFW was recorded in Sorg-23 by 0.197 g, Sorg-14 by 0.195 g, and Sorg-67 by 0.189 g (Supporting Table 2). Similarly, under T3, RFW was also significantly improved in Sorg-62 by 0.291 g, Sorg-43 by 0.279, Sorg-52 by 0.274, and Sorg-39 by 0.273. In contrast, SFW decreased with the increase in drought stress. Under controlled conditions, the highest gain in SFW was recorded in Sorg-43 by 0.818 g, followed by Sorg-39, Sorg-62, and Sorg-66, respectively. Under T2, the highest reduction in SFW was recorded in Sorg-52 by 0.056 g, Sorg-33 by 0.078 g, and Sorg-46 by 0.107 g. While under T3, SFW reached to its maximum reduction by 0.049 g in Sorg-52, 0.078 g in Sorg-56, and 0.072 g in Sorg-57 (Supporting Table 2).

Root dry weight (RDW) increased with the increase in drought stress intensity (Supporting Table 2). The maximum gain in RDW under controlled conditions was observed in Sorg-16 by 0.061 g, Sorg-8 by 0.055 g, and Sorg-41 by 0.054 g. Under T2, Sorg-41, Sorg69, and Sorg-45 gained maximum RDW by 0.061, 0.057 g, and 0.059 g, respectively. Sorg-65, Sorg-45, and Sorg-29 showed maximum gain in RDW under T3 by 0.084, 0.075, and 0.079 g, respectively (Supporting Table 2). However, SDW reduced across all treatments. Under controlled conditions, the highest reduction in SDW was observed in Sorg-17 by 0.058 g, Sorg-6 by 0.079 g, Sorg-33 by 0.078 g, and Sorg-26 by 0.077 g. Under T2 drought level, SDW decreased by 0.017, 0.030, and 0.031 g in Sorg-52, Sorg-48, and Sorg-33, respectively. At T3, SDW further reduced by 0.007, 0.010, and 0.012 g in Sorg-57, Sorg-52, and Sorg-56, respectively (Supporting Table 2).

### 3.3. Effect of Treatments on Physiological and Fodder Quality Traits

CC significantly decreased in all genotypes with the increase in drought stress levels (Supporting Table 3). Under controlled conditions, the highest CCs were measured in Sorg-12, Sorg-55, Sorg-52, and Sorg-3 by 53.6, 52.2, 50.4, and 50.7 μg cm^−2^, respectively (Supporting Table 3), while the lowest CCs were recorded in Sorg-23 by 36.9 μg cm^−2^, Sorg-21 by 42.2 μg cm^−2^, and Sorg-9 by 40 μg cm^−2^. Under T2, the highest decrease in CC was recorded by 30.5, 34.2, and 34.2 μg cm^−2^ in Sorg-23, Sorg-70, and Sorg-9, respectively. Reduction in CC was even more profound under T3, where the highest decrease was recorded in Sorg-23 by 23.4, Sorg-57 by 28.2, and Sorg-5 by 28.1 μg cm^−2^ (Supporting Table 3).

Under controlled conditions, the highest CP content were observed in Sorg-27, Sorg-29, Sorg-14, and Sorg-47 by 29.6%, 28.9%, 28.5%, and 29.2%, respectively. However, with the increase in drought stress intensity, CP significantly decreased across all the genotypes except few (Supporting Table 3). The highest reduction was recorded in Sorg-28 by 17.6%, Sorg-42 by 18%, and Sorg-39 by 18.3%. Moreover, Sorg-29, Sorg-14, Sorg-62, and Sorg-50 maintained high levels of CP by 27%, 26.7%, 26.7%, and 26.4%, respectively. Under 10% PEG stress (T3), the lowest CP was recorded in Sorg-68 by 15.4%, Sorg-60 by 16.1%, and Sorg-25 by 16.5%, respectively, while the highest was recorded by 23.1%, 22.1%, 22.5%, and 21.5% in Sorg-47, Sorg-70, Sorg-31, and Sorg-50 (Supporting Table 3).

ADF significantly varied among the genotypes and treatments. Under controlled conditions, the highest ADFs were recorded in Sorg-5, Sorg-67, Sorg-6, Sorg-2, and Sorg-69 by 23%, 21%, 19.9%, 19.3%, and 19.6%, respectively (Supporting Table 3). The lowest ADFs were recorded in Sorg-18, Sorg-26, Sorg-27, and Sorg-55 by 6.5%, 7%, 6.5%, and 7.5%, respectively. Under T2, the highest ADF levels were recorded in Sorg-5 by 20.4%, Sorg-67 by 18.2%, and Sorg-69 by 17.7%, and the lowest were recorded by 3.7%, 4.5%, and 4% in Sorg-18, Sorg-26, and Sorg-27, respectively. Under T3, the highest reduction in ADF values was recorded in Sorg-18 by 1.7%, Sorg-26 by 2.3%, and Sorg-28 by 2.4% with the lowest reduction in Sorg-5 by 21.9%, Sorg-67 by 16.1%, and Sorg-69 by 14.9% (Supporting Table 3).

NDF increased under stress, ranging from 32% to 49% in T1, 25%–43% in T2, and 14%–36% in T3. Among the genotypes, Sorg-66 exhibited the highest NDF values, while Sorg-60 recorded the lowest. Ash content also increased with stress, ranging from 14% to 25% in T1, 10%–21% in T2, and 6%–17% in T3; Sorg-66 had the highest ash content, whereas Sorg-56 showed the greatest reduction (23%) (Supporting Table 3). Crude fiber ranged from 3.6% to 5.5% in T1, 2.7%–4.6% in T2, and 1.1%–3.5% in T3. Sorg-66 again outperformed other genotypes, while Sorg-53 had the largest decrease (57%) (Supporting Table 3).

Ash content also varied significantly among the treatments and genotypes. Under controlled conditions, the highest ash contents were recorded in Sorg-18, Sorg-26, Sorg-28, and Sorg-30 by 24.6%, 25.4%, 25.2%, and 24.6%, respectively, and the lowest ash contents were recorded by 14.8%, 14.4%, and 14.9% in Sorg-63, Sorg-62, and Sorg-43, respectively (Supporting Table 3). Under T2 conditions, the highest ash contents were recorded in Sorg-28 by 21.8%, Sorg-30 by 21.5%, and Sorg-50 by 20.8%. Under T3 conditions, the highest Ash contents were recorded in Sorg-26 by 17.3%, Sorg-28 by 17.3%, and Sorg-30 by 16.3% (Supporting Table 3).

Under controlled conditions, the highest increase in CF content was observed in Sorg-50, Sorg-59, Sorg-52, Sorg-19, and Sorg-22 by 5.5%, 5.1%, 5%, 5%, and 4.91%, respectively, and the lowest was observed in Sorg-67 by 3.6%, Sorg-68 by 3.6%, and Sorg-38 and Sorg-61 by 3.7% (Supporting Table 3). Under T2, the CF content varied with the highest in Sorg-50 by 5%, Sorg-59 by 4%, Sorg-53 by 4%, and Sorg-52 by 4%. The lowest CF content was recorded by 2.9% in Sorg-61 and Sorg-62. Under T3, the highest CF content was recorded in Sorg-50 and Sorg-22 by 3.5% and 2.9%, respectively (Supporting Table 3).

HCN levels increased with increasing stress severity, ranging from 57.9 to 695 ppm in T1, 86.4–723 ppm in T2, and 107–779 ppm in T3. Sorg-66 showed the highest HCN accumulation, and Sorg-53 demonstrated the greatest percentage increase (68%) (Supporting Table 3). RWC ranged from 82% to 96% in T1, 74%–93% in T2, and 78%–91% in T3. Sorg-66 maintained the highest RWC, while Sorg-60 showed the lowest. PC also increased under stress, ranging from 523 to 1006 μmol mg^−1^ FW in T1, 491–1125 μmol mg^−1^ FW in T2, and 591–1232 μmol mg^−1^ FW in T3, with Sorg-66 recording the highest accumulation (Supporting Table 3).

RWC decreased consistently with the increase in drought stress. Under controlled conditions, highest RWC was recorded in Sorg-67, Sorg-62, Sorg-65, Sorg-2, and Sorg-64 by 96%, 95%, 95.2%, 94.4%, and 94.3%, respectively, whereas the lowest RWC was observed in Sorg-9 by 82.1%, Sorg-16 by 82.8%, and Sorg-34 by 82.6%. Under T2, maximum water content retained in Sorg-62 by 93.3%, Sorg-65 and Sorg-67 by 93.6%, and Sorg-64 by 92% (Supporting Table 3). The lowest RWC retained in Sorg-52 by 77%, Sorg-31 by 78%, and 60 by 78.3%. Under T3, the highest RWC was recorded in Sorg-62 by 91.5%, Sorg-65 by 91.8%, and Sorg-67 by 91.6%. The lowest RWC under T3 was recorded in Sorg-16 by 78.2%, Sorg-9 by 79%, and Sorg-34 by 78.4% (Supporting Table 3).

PC increased progressively with the increase in drought stress levels. Under controlled conditions, the highest increase in PC was recorded in Sorg-14, Sorg-65, and Sorg-3 by 1006.4, 956.6, and 940.7 μmol g^−1^. In T2, the highest increase in PC was recorded by 1095.3, 1125.3, and 1050.2 μmol g^−1^ in Sorg-43, Sorg-53, and Sorg-26. In T3, the highest accumulation of PC was recorded in Sorg-14, Sorg-42, Sorg-2, and Sorg-9 by 1232.8, 1189.4, 1185.3, and 1184.8 μmol g^−1^, respectively (Supporting Table 3).

### 3.4. Principal Component Analysis

**Figure 1:**
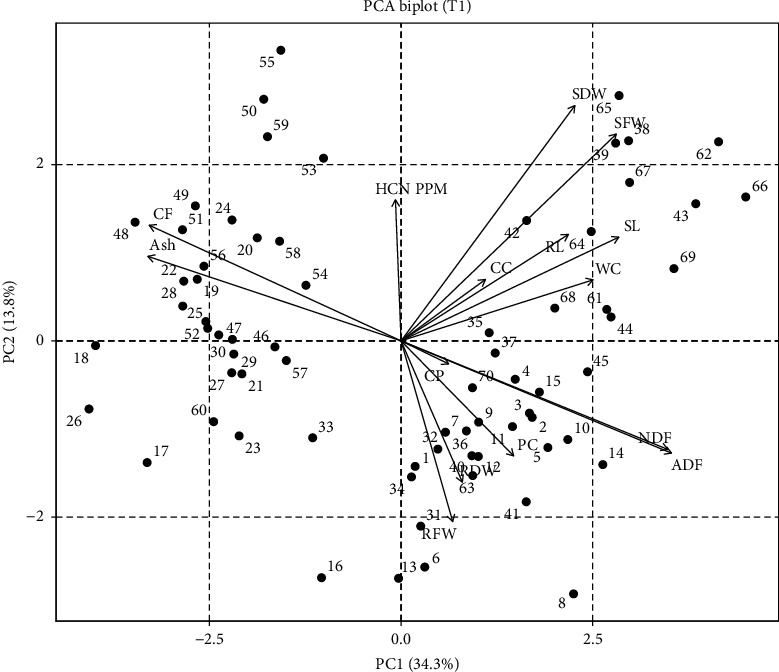
Principal component analysis (PCA) biplot showing the distribution of 70 sorghum genotypes under control conditions (T1).

ADF showed the highest eigenvalues, followed by ash content, CF, and CC, whereas RWC, PC, and SL had minimum eigenvalues, respectively, as shown in Table 2. PCA of 70 genotypes for seedling characteristics related to yield traits in T1 showed most of the traits under observation fell in quadrants I and IV, stem fresh weight, stem dry weight, SL, RL, RWC, ADF, NDF, and root dry weight. Sorg-38, Sorg-42, Sorg-6, and Sorg-69 fell in quadrate I, while Sorg-12, Sorg-63, Sorg-6, Sorg-9, and Sorg-70 fell in quadrate IV, representing that these genotypes can be selected for further selection (Figure 1).

**Figure 2:**
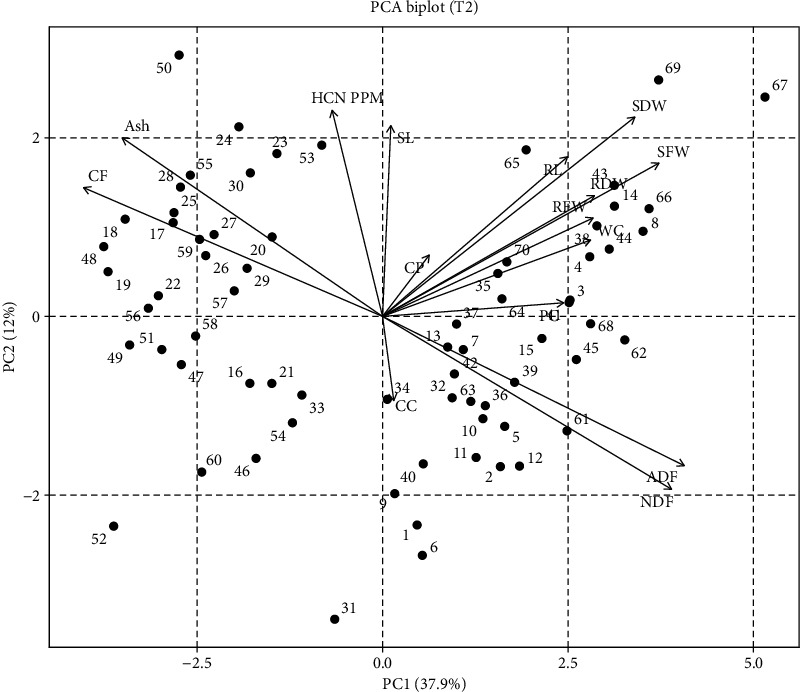
Principal component analysis (PCA) biplot showing the distribution of 70 sorghum genotypes under 5% PEG-induced drought stress (T2).

PCA for T2 represented that most of the traits fell in quadrate I and the rest in quadrates II and IV. Stem fresh weight and dry weight, root dry weight and fresh weight, RWC, RL, and SL fell in quadrate I (Supporting Table 4), while ash content, CF, and HCN fell in quadrate II, and ADF, NDF, and PC fell in quadrate IV. Sorg-66, Sorg-3, Sorg-65, Sorg-38, and Sorg-8 fell in quadrate I, while Sorg-30, Sorg-55, Sorg-25, Sorg-28, and Sorg-29 fell in quadrate II, and Sorg-39, Sorg-61, Sorg-15, and sorg-42 fell in quadrate IV, representing these genotypes to be effective for selection of the traits mentioned (Figure 2).

**Figure 3:**
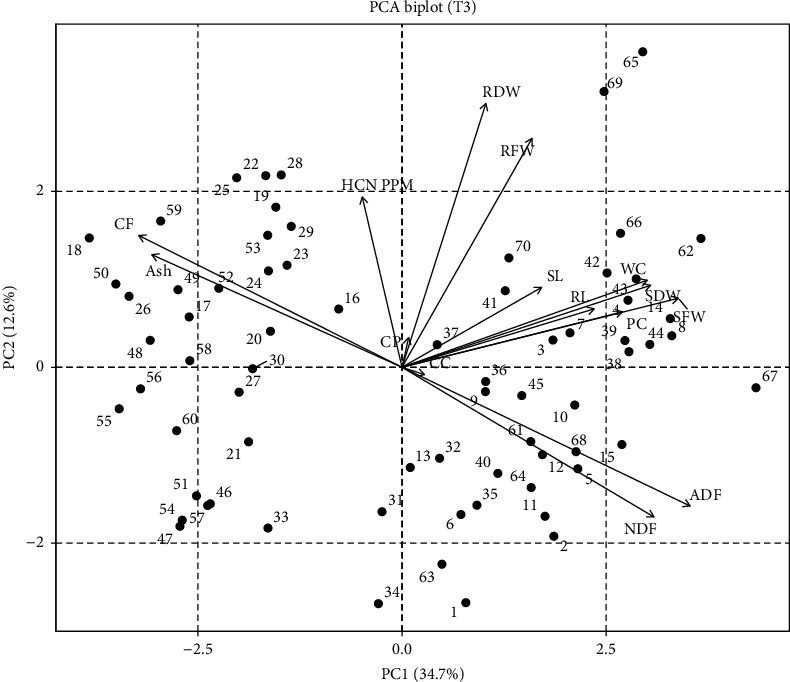
Principal component analysis (PCA) biplot representing the distribution of 70 sorghum genotypes under 10% PEG-induced drought stress (T3).

PCA for T3 showed that genotypes Sorg-66, Sorg-13, and Sorg-67 fell in quadrate I, representing their strength for seedling traits as well as for some quality traits, while Sorg-27, Sorg-5, and Sorg-45 fell in quadrate IV and represented the strength for CC and ADF; Sorg-28, Sorg-59, and Sorg-52 fell in quadrate II, representing the strength of HCN, ash content, CP, and CF as mentioned in Figure 3.

### 3.5. Pearson Correlation Analysis Among Traits Under All Treatments

**Figure 4:**
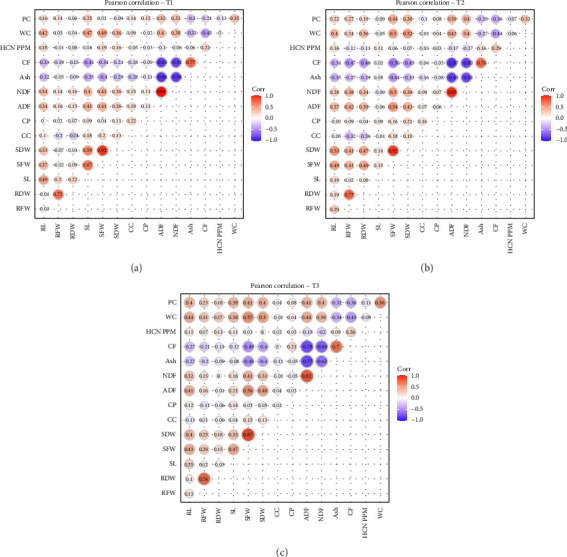
Pearson correlation analyses performed between traits across all treatments including T1 (control) (a), T2 (PEG 5%), and (b) and T3 (PEG 10%) (c).

Pearson correlation analyses were performed to evaluate the relationship among the traits across different treatments. Significant positive and negative correlations were observed among the studied traits. Under controlled conditions, strong positive correlations were observed among SDW and SFW (*r* = 0.92), SFW and SL (*r* = 0.67), RDW and RFW (*r* = 0.72), NDF and ADF (*r* = 0.99), and RWC and SFW (*r* = 0.49). NDF and ADF showed a strong negative correlation with CF (*r* = −0.81) and (*r* = −0.83), respectively. RWC and SL also showed a weak negative correlation with CF (*r* = −0.45) and (*r* = −0.43), respectively (Figure 4(a)).

Under 5% PEG (T2), drought stress NDF had a strong positive correlation with ADF (*r* = 0.95), SDW had a strong positive correlation with SFW (*r* = 0.92), and RDW had a strong positive correlation with RFW (*r* = 0.77). NDF had a slight positive correlation with SFW (*r* = 0.50), and ADF had a slight positive correlation with SFW (*r* = 0.54). SDW had a positive correlation with RL (*r* = 0.53) and RWC (*r* = 0.52). CF had a strong negative correlation with ADF (*r* = −0.87) and NDF (*r* = −0.83). CF also showed a slight negative correlation with RFW (*r* = −0.47), RDW (*r* = −0.46), SFW (*r* = −0.56), and RWC (*r* = −0.44) (Figure 4(b)). Under 10% PEG (T3), drought stress SDW had a strong positive correlation with SFW (*r* = 0.87), NDF had a strong positive correlation with ADF (*r* = 0.82), and RDW had a strong positive correlation with RFW (*r* = 0.76). Moreover, SFW had a slight positive correlation with SL (*r* = 0.47), ADF (*r* = 0.56), and RWC (*r* = 0.57). NDF and ADF had a strong negative correlation with CF (*r* = −0.69) and (*r* = −0.78), respectively (Figure 4(c)).

### 3.6. Evaluation and Screening of Selected Sorghum Genotypes Under Drought Stress at Maturity

#### 3.6.1. ANOVA

ANOVA depicted significant differences among 20 genotypes of sorghum at the maturity stage for fodder quality, yield, and physiological parameters related to drought under observation (Table 3). Genotypes (G), treatments (T), and genotype × treatment interaction were significant for most of the traits. Genotypic variation was found highly significant for all the traits such as PH, NOL, LA, CP, ash, CF, TDN, GFY, NDF, ADF, PC, CC, RWC, and HCN. It indicates that the selected genotypes possess significant genetic variability.

#### 3.6.2. Mean Performance of Selected Sorghum Genotypes at Maturity Under Drought Stress

The mean performance of 20 sorghum genotypes under normal and drought conditions revealed significant variations across all studied traits, demonstrating the impact of environmental stress on plant growth and nutritional quality. Under drought conditions, a general decline was observed in key agronomic traits, including the NOL (reduced from 13 to 24 to 12–18), PH (decreased from 114.26–210.17 cm to 91.46–273.22 cm), and LA (which dropped from 257.7–463.7 cm^2^ to 91.49–273.4 cm^2^). Similarly, reductions were recorded in CP content (6.51%–13.8% under normal conditions to 3.56%–11.4% under drought), ash content (8.4%–16.3% to 4.1%–16.8%), CF (28.1%–40.2% to 15.7%–31%), TDN (59.3%–78.3% to 55.1%–73.5%), and GFY (388.8–784.4 g to 326.3–698.8 g). Additionally, NDF declined from 16.1%-32.1% to 9.2%–32.1%, CC declined from 31.8–44.1 μg cm^−2^ to 27.1–42.4 μg cm^−2^, and RWC declined from 74%–93.4% to 73.5%–86.1% (Supporting Table 6).

On the other hand, certain biochemical traits exhibited an increase under drought stress, likely as part of the plant's adaptive mechanisms. ADF increased from 16.5%–32.2% to 18.5%–33%, while PC rose from 523.3 to 695.6 μmol mg^−1^ FW under normal conditions to 466.2–770.1 μmol mg^−1^ FW under drought stress. HCN content, which indicates potential toxicity risks, also increased, ranging from 72.4 to 330 ppm under normal conditions to 89.3–358 ppm under drought conditions (Supporting Table 6).

Among the studied genotypes, Sorg-66, Sorg-41, and Sorg-70 demonstrated resilience under drought stress, maintaining relatively higher values for key traits, whereas Sorg-55 and Sorg-60 exhibited the highest reductions in multiple parameters. Notably, the highest percentage decreases were observed in LA (46.1%, Sorg-55), CP (45.2%, Sorg-47), ash content (49.1%, Sorg-55), and CF (49.2%, Sorg-55), while HCN (36.6%, Sorg-13) and proline (13.3%, Sorg-13) exhibited the highest increases. These findings highlight the significant impact of drought on sorghum growth and nutritional value and provide valuable insights for selecting drought-tolerant genotypes for future breeding programs aimed at improving resilience and productivity under water-limited conditions (Supporting Table 6).

#### 3.6.3. Principal Component Analysis

**Figure 5:**
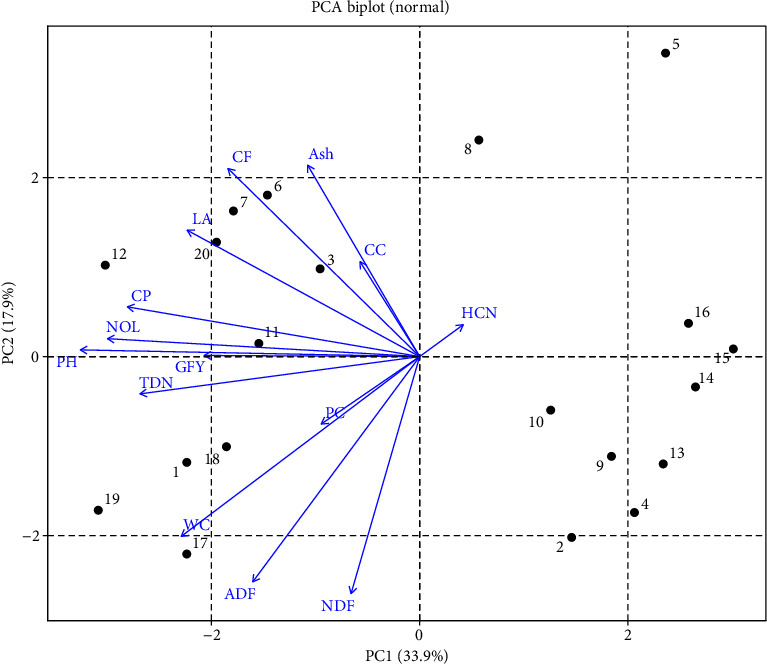
Principal component analysis (PCA) biplot of 20 selected sorghum genotypes evaluated under normal (nonstress) field conditions. Principal components PC1 and PC2 accounted for 33.9% and 17.9% of the total variation, respectively.

ADF showed the highest eigenvalues, followed by ADF, GFY, and HCN, whereas PH, LA, and CP had minimum eigenvalues, respectively, as shown in Table 4. PCA of 20 genotypes at maturity for yield traits in normal conditions showed all the traits under observation fell in quadrates I and IV. Sorg-60, Sorg-62, Sorg-66, and Sorg-5 fell in quadrate I, while Sorg-6, Sorg-23, Sorg-18, Sorg-34, Sorg-37, and Sorg-67 fell in quadrate IV representing that these genotypes can be selected for further selection (Figure 5).

**Figure 6:**
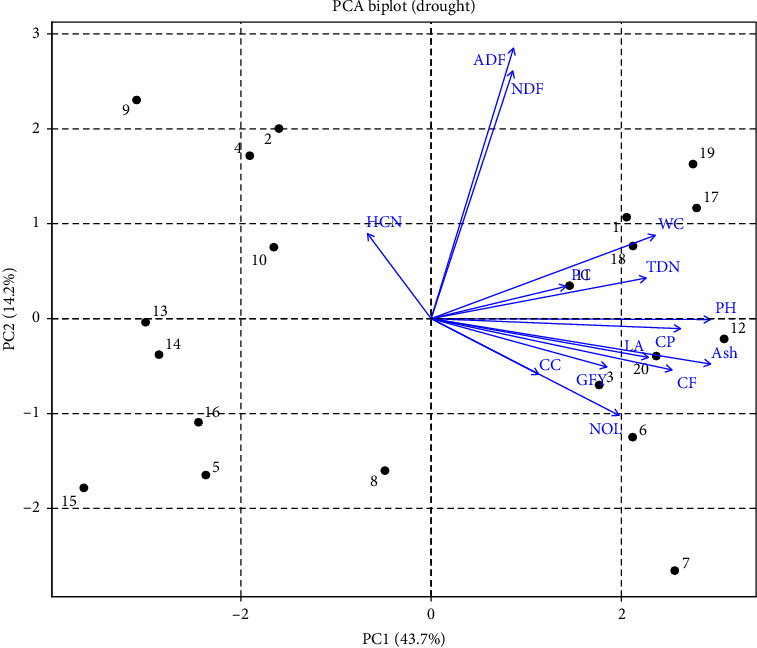
Principal component analysis (PCA) biplot of 20 selected sorghum genotypes evaluated under drought (stress) field conditions. Principal components PC1 and PC2 accounted for 43.7% and 14.2% of the total variation, respectively.

PCA for drought represented that most of the traits fell in quadrate I and the rest in quadrate IV, except that HCN fell in quadrate II. Water content, PC, PH, ADF, NDF, TDN, and CP fell in quadrate I, while ash content, CF, NOL, and CC fell in quadrate IV. Sorg-5, Sorg-67, Sorg-62, Sorg-60, and Sorg-37 fell in quadrate I, while Sorg-2, Sorg-4, Sorg-10, Sorg-9, and Sorg-13 fell in quadrate II, and Sorg-41, Sorg-70, Sorg-13, and Sorg-23 fell in quadrate IV, representing these genotypes to be effective for selection of the traits mentioned (Figure 6).

#### 3.6.4. Pearson Correlation Analyses of Selected Genotypes at Field Conditions

**Figure 7:**
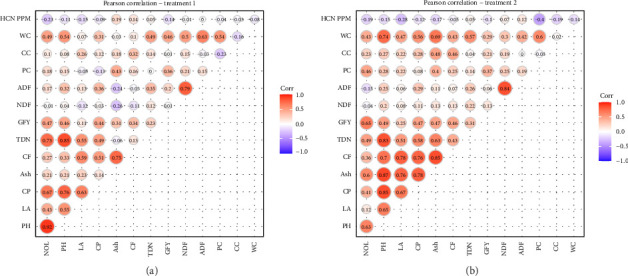
Pearson correlation analyses performed on selected 20 genotypes under normal (Treatment 1) (a) and drought (Treatment 2) (b) at field conditions.

Correlation analyses revealed significant, strong positive and negative correlations among the traits under field conditions. Under normal field conditions (T1), ADF showed a strong positive correlation with NDF (*r* = 0.79). CF had strong positive correlations with CP (*r* = 0.51) and LA (*r* = 0.59), while LA also correlated positively with CP (*r* = 0.63). TDN showed strong positive associations with PH (*r* = 0.85), NOL (*r* = 0.73), and WC (*r* = 0.63). NOL exhibited positive correlations with GFY (*r* = 0.47) and CP (*r* = 0.67). HCN showed a weak negative correlation with NOL (*r* = −0.23) and with LA (*r* = −0.15) (Figure 7(a)). Under drought field conditions (T2), ADF showed a strong positive correlation with NDF (*r* = 0.84). Ash had a strong positive correlation with CF (*r* = 0.85) and PH (*r* = 0.87). CF was also strongly correlated with CP (*r* = 0.76) and moderately with LA (*r* = 0.78). PH showed positive correlations with TDN (*r* = 0.83) and NOL (*r* = 0.63). HCN exhibited weak negative correlations with LA (*r* = −0.28) and NOL (*r* = −0.19) (Figure 7(b)).

## 4. Discussion

Abiotic stresses, particularly drought, are known to adversely affect key morphological and physiological traits in plants including, SL, SFW, SDW, RWC, Ash, and CC [19]. In this study, 70 sorghum genotypes at seedling stage were exposed to different levels of drought stress (0%, 5%, and 10%), which significantly impacted multiple traits. Notably, drought stress led to reductions in RL (e.g., a 28% decrease in Sorg-56 at 5%), SL, biomass, and CC (e.g., a 37% decrease in Sorg-53 at 10%). Interestingly, RWC showed an increase of 82% at 0% and 93% at 10% drought stress, suggesting a possible compensatory mechanism in some genotypes. These results are in agreement with the results reported by [10, 20]. These reductions are primarily driven by limited water uptake and osmotic stress, which disrupt cellular expansion and reduce turgor pressure, ultimately inhibiting growth. RL increased under drought, likely as an adaptive response to access deeper soil moisture, which aligns with known drought survival strategies in fodder and cereals. [21]. However, a few genotypes, such as Sorg-12, Sorg-55, and Sorg-60, exhibited notable resilience, showing improvements in morphological and physiological traits across all levels of drought stress at the seedling stage. Moreover, CP, ADF, and NDF showed significant increase of 27.4% at 5%, 21.8% at 10%, and 43% at 5% drought stress, respectively. Genotype Sorg-27 exhibited the highest increase in CP, while Sorg-5 showed the greatest increase in ADF, and Sorg-66 recorded the maximum increase in NDF. We also observed decreases in CP, ADF, and NDF in genotype Sorg-53 by 41% and 73% and in Sorg-60 by 33%, respectively. Similarly, significant variations in ash content were also observed, with Sorg-66 showing the highest increase and Sorg-56 exhibiting the greatest decrease, with a reduction of 23%. These findings are consistent with the results reported by [22–25]. Variability in CP content under drought both increases and decreases suggests genotype-dependent metabolic adjustments. While some genotypes may synthesize stress-related proteins, others may suffer protein degradation due to stress severity. Similarly, forage quality traits like NDF, ADF, and ash content showed mixed responses, indicating that drought alters both structural and nutritional aspects of biomass [10, 26–28]. Both CF and HCN levels increased across all drought stress levels. At 5% and 10% drought stress, CF increased by 4.6% and 3.5%, respectively, while HCN content increased by 723 and 779 ppm. Genotype Sorg-66 exhibited the maximum increase in these traits, including CF and HCN contents. In contrast, Sorg-53 showed the greatest reduction in CP content (57%) and the highest decrease in HCN content (68%). An increase in HCN under drought is concerning, as it may pose toxicity risks. This stress-induced dhurrin accumulation reflects a defense mechanism, yet highlights the need for low-HCN genotypes. Correlation analyses at seedling stage revealed several strong positive and negative correlations, indicating the dependency of traits on each other. For example, the increase in SL directly influences SFW showing significant increase and developing resistance to drought stress. Similarly, fodder quality traits also depend on each other under both normal and drought conditions. For example, the increase in ADF and NDF contents decreases CF which is an essential fodder nutrient element required for easy digestion.

Under field conditions, the 20 selected sorghum genotypes showed a pronounced response to drought, confirming the trends observed during the seedling stage. The reduction in PH (from 114.26 to 91.46 cm), NOL (from 24 to 18), and LA (from 257.7 to 91.49 cm^2^) under drought conditions reflects the genotypes' limited capacity to maintain cellular growth and expansion when water is scarce. This is likely due to reduced cell turgor, stomatal closure, and lower photosynthetic efficiency under water-limited environments. Diminished leaf number and area further restrict light interception and carbon assimilation, exacerbating growth reductions. In terms of forage quality, drought stress led to significant variation in nutritional traits. For example, the reduction in TDN (from 78.3% to 55.1%), CP (from 6.51% to 3.56%), and ash content (from 8.4% to 4.1%) can be attributed to impaired nutrient uptake, altered nitrogen metabolism, and reduced biosynthesis of structural and metabolic proteins [29, 30]. Moreover, reductions were also observed in NDF, CC, and water content, which decreased from 16.1% to 9.2%, 31.8 μg cm^−2^ to 27.1 μg cm^−2^, and 93.4% to 86.1%, respectively. On the other hand, ADF may increase (from 16.5% to 18.5%) under drought due to higher lignin and cellulose deposition as part of the plant defense response. Such structural reinforcement, while enhancing drought resistance, can reduce digestibility and forage palatability [31]. Interestingly, the increase in HCN (89.3–358 ppm) content under drought is consistent with stress-induced activation of cyanogenic glycoside pathways. Dhurrin accumulation, which serves as a chemical defense, is typically triggered by osmotic stress and may pose a toxicity risk for livestock if levels exceed the safe threshold [32, 33]. Therefore, evaluating both biomass productivity and safety parameters like HCN is crucial for selecting suitable genotypes for fodder purposes under climate stress [22, 24, 34–36].

PCA further revealed strong correlations among key traits under drought, helping to identify tolerant genotypes with favorable quality and safety profiles. The clustering of SFW, SDW, RL, and SL indicated a strong positive correlation among these traits, suggesting that genotypes capable of maintaining shoot and root growth under drought are likely to sustain biomass accumulation. This is a valuable indicator of drought tolerance, as it reflects the plant's ability to maintain water and nutrient uptake through a more developed root system and allocate resources efficiently to shoot growth (Figure 1) [23, 25]. Conversely, ash and CF contents were negatively correlated with ADF and NDF, implying that genotypes with higher ash and CF tend to have lower levels of structural carbohydrates like cellulose and lignin [12, 37–40]. This relationship is important from a forage quality perspective, as lower ADF and NDF levels generally enhance digestibility and feed efficiency, while higher ash may indicate better mineral content (Figures 1 and 2) [41]. HCN content displayed a strong positive correlation with SL, suggesting that more vigorous shoot elongation under stress may inadvertently enhance cyanogenic activity (Figures 2 and 4) [42]. However, HCN was negatively correlated with both CC and water content, indicating that genotypes with higher HCN accumulation may suffer more from drought-induced reductions in photosynthetic efficiency and water retention [43]. These multivariate relationships emphasize the value of PCA in distinguishing drought-resilient genotypes with favorable agronomic and forage quality traits. Genotypes such as Sorg-60, Sorg-66, and Sorg-7 not only maintained biomass and nutritional quality under drought but also exhibited lower HCN accumulation, making them ideal candidates for fodder production in water-limited environments. The findings of this study have practical relevance beyond Pakistan, as the identified sorghum genotypes with improved drought tolerance, reduced HCN levels, and enhanced forage quality can be effectively utilized in arid and semiarid regions. These results are particularly applicable to other drought-prone sorghum-growing areas of South Asia, Sub-Saharan Africa, and the Middle East, where forage shortages and water scarcity remain critical challenges. Hence, the outcomes contribute to broader efforts in developing resilient forage production systems under climate stress.

## 5. Conclusion

This study identified significant genetic diversity among 70 sorghum genotypes for drought tolerance and low HCN content, critical for sustainable fodder production in Pakistan. Genotypes such as Sorg-60, Sorg-66, and Sorg-7 showed superior drought resilience, forage yield, and fodder quality, while others like Sorg-53 and Sorg-56 were more sensitive to stress. Field evaluation of selected genotypes confirmed their performance under drought, with some maintaining favorable physiological and biochemical traits and relatively low HCN levels. PCA highlighted key traits associated with drought tolerance. These findings can guide breeding programs focused on developing high-yielding, stress-resilient sorghum cultivars. Future work should explore multilocation field trials and genomic tools to accelerate cultivar development.

## Figures and Tables

**Table 1:** Analysis of variance of 70 sorghum genotypes for seedling traits under 3 treatments for drought stress.

SOV	DF	RL	RFW	RDW	SFW	SDW	SL	ADF	NDF	CF	CP	HCN	CC	RWC	PC	Ash
Genotypes (G)	69	72,544.6^∗∗^	286.6^∗∗^	55.4^∗∗^	809.3^∗∗^	365.6^∗∗^	35,780.2^∗∗^	61,505.5^∗∗^	66,168.0^∗∗^	176.9^∗∗^	6407.3^∗∗^	9821.2^∗∗^	27,134.1^∗∗^	706.8^∗∗^	721.3^∗∗^	25,094.4^∗∗^
Treatments (T)	2	803,646.0^∗∗^	16,959.9^∗∗^	2582.3^∗∗^	14,955.3^∗∗^	21,584.4^∗∗^	2,305,682.0^∗∗^	285,402.0^∗∗^	2,312,939.0^∗∗^	348.8^∗∗^	808,826.0^∗∗^	20,510.7^∗∗^	2,424,711.0^∗∗^	36,705.5^∗∗^	20,785.5^∗∗^	996,974.0^∗∗^
Interaction (G × T)	138	3513.7^∗∗^	102.9^∗∗^	15.4^∗∗^	66.5^∗∗^	78.4^∗∗^	19,429.1^∗∗^	622.1^∗∗^	3733.2^∗∗^	56.0^∗∗^	2184.4^∗∗^	79.8^∗∗^	2253.4^∗∗^	697.4^∗∗^	201.9^∗∗^	625.7^∗∗^

Abbreviations: ADF = acid detergent fiber, CC = chlorophyll content, CF = crude fiber, CP = crude protein, HCN = hydrogen cyanide, NDF = neutral detergent fiber, PC = proline content, RDW = root dry weight, RFW = root fresh weight, RL = root length, RWC = relative water content, SDW = shoot dry weight, SFW = shoot fresh weight, and SL = shoot length.

^∗^Significant at 5% probability level.

^∗∗^Significant at 1% probability level.

**Table 2:** Eigenvalues and variability percentage of 70 sorghum genotypes for different traits at seedling stage.

	ADF	ASH	CF	CC	CP	HCN	NDF	RDW	RFW	RL	SDW	SFW	SL	PC	WC
Eigenvalue	5.14	2.06	1.15	1.09	1.83	0.94	0.69	0.63	0.50	0.30	0.24	0.17	0.13	0.04	0.009
Variability (%)	34.3	13.8	12.2	7.7	7.3	6.3	4.6	4.2	3.4	2.0	1.7	1.2	0.9	0.3	0.1

*Note:* CP= crude fiber, HCN=hydrogen cyanide.

Abbreviations: ADF = acid detergent fiber, CC = chlorophyll content, CF = crude fiber, NDF = neutral detergent fiber, PC = proline content, RDW = root dry weight, RFW = root fresh weight, RL = root length, SDW = shoot dry weight, SFW = shoot fresh weight, SL = shoot length, WC = water content.

**Table 3:** Analysis of variance of 20 selected sorghum genotypes for maturity traits.

SOV	DF	NOL	PH	LA	CP	Ash	CF	TDN	GFY	NDF	ADF	PC	CC	RWC	HCN
Genotypes (G)	19	50.84^∗∗^	795.50^∗∗^	81.90^∗∗^	397.60^∗∗^	605.20^∗∗^	1080.30^∗∗^	2287.80^∗∗^	459.50^∗∗^	2433.01^∗∗^	1824.10^∗∗^	132.70^∗∗^	740.20^∗∗^	1111.10^∗∗^	499.30^∗∗^
Treatments (T)	1	363.50^∗∗^	479.80^∗∗^	2118.40^∗∗^	2174.10^∗∗^	188.40^∗∗^	43,649.00^∗∗^	5116.40^∗∗^	608.30^∗∗^	266.20^∗∗^	1246.30^∗∗^	324.80^∗∗^	11,299.80^∗∗^	8685.30^∗∗^	372.60^∗∗^
Interaction G × T	19	6.34^∗∗^	115.70^∗∗^	1.60^∗^	5.90^∗∗^	244.06^∗∗^	74.90^∗∗^	3.06^∗∗^	6.70^∗∗^	127.45^∗∗^	29.81^∗∗^	19.04^∗∗^	123.40^∗∗^	163.70^∗∗^	1.04^∗^

Abbreviations: ADF = acid detergent fiber, CC = chlorophyll content, CF = crude fiber, CP = crude protein, GFY = green forage yield, HCN = hydrogen cyanide, LA = leaf area, NDF = neutral detergent fiber, NOL = number of leaves, PC = proline content, PH = plant height, RWC = relative water content, and TDN = total digestible nutrients.

^∗^Significant at 5% probability level.

^∗∗^Significant at 1% probability level.

**Table 4:** Eigenvalues and variability percentage of 20 sorghum genotypes for different traits at the maturity stage.

	ADF	GFY	CF	HCN	NDF	NOL	PC	TDN	WC	ASH	CC	CP	LA	PH
Eigenvalue	4.74	2.50	1.85	1.40	1.05	0.76	0.70	0.34	0.21	0.17	0.13	0.43	0.041	0.007
Variability (%)	33.9	17.9	13.3	10	7.5	5.5	5	2.5	1.6	1.3	1	0.3	0.3	0.1

*Note:* HCN=hydrogen cyanide.

Abbreviations: ADF = acid detergent fiber, CC = chlorophyll content, CF = crude fiber, CP = crude protein, GFY = green forage yield, LA = leaf area, NDF = neutral detergent fiber, N.O.L = number of leaves, PC = proline content, PH = plant height, TDN = total digestible nutrients, WC = water content.

## Data Availability

The data that support the findings of this study are available from the corresponding author upon reasonable request.

## Supporting Information

Supporting Information 1 Supporting Material File 1 contains detailed lists and analytical data of sorghum genotypes evaluated under drought stress. It includes the list of 70 genotypes screened at the seedling stage (Supporting Table 1).

Supporting Information 2 Supporting Material File 2 provides quantitative data on the performance of sorghum genotypes under varying drought stress levels. It includes mean values of morphological and physiological traits, as well as correlation analyses among these traits, supporting the main findings of the study.
