# Supplementary material for: Comprehensive Assessment of Local and Exotic Sorghum Genotypes for Forage Production and Quality Under Drought Conditions
Source: Scientifica (Cairo). 2025 Nov 28;2025:9158280. doi: 10.1155/sci5/9158280 (PMC12680474; doi:10.1155/sci5/9158280)
Supplement: Supporting Information 1 — Supporting Material File 1 contains detailed lists and analytical data of sorghum genotypes evaluated under drought stress. It includes the list of 70 genotypes screened at the seedling stage (Supporting Table 1). [file 9158280.f1.docx]

**Supplementary Table 1.** List of 70 genotypes screened at seedling stage under all three drought stress levels.

| **Sr.no.** | **Genotypes** | **code** | **Sr.no.** | **genotypes** | **code** | **Sr.no.** | **genotypes** | **code** | **Sr.no.** | **genotypes** | **Code** | **Sr. no.** | **genotypes** | **Code** | **Sr.no.** | **genotypes** | **Code** | **Sr.no.** | **genotypes** | **code** |
| --- | --- | --- | --- | --- | --- | --- | --- | --- | --- | --- | --- | --- | --- | --- | --- | --- | --- | --- | --- | --- |
| **1** | GM-726 | Sorg-1 | **11** | GP-46 | Sorg-11 | **21** | GM-726 | Sorg-21 | **31** | U-59 | Sorg-31 | **41** | P-4 | Sorg-41 | **51** | U-51 | Sorg-51 | **61** | P-7 | Sorg-61 |
| **2** | S-145 | Sorg-2 | **12** | F-04-10 | Sorg-12 | **22** | S-145 | Sorg-22 | **32** | No-6001 | Sorg-32 | **42** | P-9 | Sorg-42 | **52** | U-52 | Sorg-52 | **62** | P-10 | Sorg-62 |
| **3** | PARC.SV-4 | Sorg-3 | **13** | NO-80230 | Sorg-13 | **23** | S-146 | Sorg-23 | **33** | No-9706 | Sorg-33 | **43** | P-15 | Sorg-43 | **53** | U-58 | Sorg-53 | **63** | P-15 | Sorg-63 |
| **4** | I-15 | Sorg-4 | **14** | SA-013-1 | Sorg-14 | **24** | S-147 | Sorg-24 | **34** | No-1563 | Sorg-34 | **44** | P-18 | Sorg-44 | **54** | U-60 | Sorg-54 | **64** | P-19 | Sorg-64 |
| **5** | I-13 | Sorg-5 | **15** | NO-1828 | Sorg-15 | **25** | S-148 | Sorg-25 | **35** | No-80230 | Sorg-35 | **45** | P-19 | Sorg-45 | **55** | U-63 | Sorg-55 | **65** | P-21 | Sorg-65 |
| **6** | F-906 | Sorg-6 | **16** | JS-2002 | Sorg-16 | **26** | S-149 | Sorg-26 | **36** | No-1862 | Sorg-36 | **46** | U-3 | Sorg-46 | **56** | U-70 | Sorg-56 | **66** | U-10 | Sorg-66 |
| **7** | SILLI | Sorg-7 | **17** | I-5 | Sorg-17 | **27** | S-150 | Sorg-27 | **37** | No-1001 | Sorg-37 | **47** | U-13 | Sorg-47 | **57** | U-101 | Sorg-57 | **67** | U-15 | Sorg-67 |
| **8** | N0-1563 | Sorg-8 | **18** | SA-013-1 | Sorg-18 | **28** | No.-8008 | Sorg-28 | **38** | SILI | Sorg-38 | **48** | U-28 | Sorg-48 | **58** | U-112 | Sorg-58 | **68** | U-44 | Sorg-68 |
| **9** | Gp-45 | Sorg-9 | **19** | Yrr-16 | Sorg-19 | **29** | No-1863 | Sorg-29 | **39** | SGD | Sorg-39 | **49** | U-45 | Sorg-49 | **59** | U-113 | Sorg-59 | **69** | U-45 | Sorg-69 |
| **10** | Yrr-I | Sorg-10 | **20** | I-4 | Sorg-20 | **30** | No-8128 | Sorg-30 | **40** | GP-51 | Sorg-40 | **50** | U-48 | Sorg-50 | **60** | U-123 | Sorg-60 | **70** | U-52 | Sorg-70 |
